# Intrinsic FGF2 and FGF5 promotes angiogenesis of human aortic endothelial cells in 3D microfluidic angiogenesis system

**DOI:** 10.1038/srep28832

**Published:** 2016-06-30

**Authors:** Ha-Rim Seo, Hyo Eun Jeong, Hyung Joon Joo, Seung-Cheol Choi, Chi-Yeon Park, Jong-Ho Kim, Ji-Hyun Choi, Long-Hui Cui, Soon Jun Hong, Seok Chung, Do-Sun Lim

**Affiliations:** 1 Department of Cardiology, Cardiovascular Center, College of Medicine, Korea University, 145 Anam-ro, Seongbuk-gu, Seoul, 02841, Republic of Korea; 2 School of Mechanical Engineering, Korea University, 145 Anam-ro, Seongbuk-gu, Seoul, 02841, Republic of Korea

## Abstract

The human body contains different endothelial cell types and differences in their angiogenic potential are poorly understood. We compared the functional angiogenic ability of human aortic endothelial cells (HAECs) and human umbilical vein endothelial cells (HUVECs) using a three-dimensional (3D) microfluidic cell culture system. HAECs and HUVECs exhibited similar cellular characteristics in a 2D culture system; however, in the 3D microfluidic angiogenesis system, HAECs exhibited stronger angiogenic potential than HUVECs. Interestingly, the expression level of fibroblast growth factor (FGF)2 and FGF5 under vascular endothelial growth factor (VEGF)-A stimulation was significantly higher in HAECs than in HUVECs. Moreover, small interfering RNA-mediated knockdown of FGF2 and FGF5 more significantly attenuated vascular sprouting induced from HAECs than HUVECs. Our results suggest that HAECs have greater angiogenic potential through FGF2 and FGF5 upregulation and could be a compatible endothelial cell type to achieve robust angiogenesis.

Neo-angiogenesis is an essential process to enhance vessel regeneration^1^,^2^. Many studies have focused on endothelial cells to explore the novel mechanisms underlying angiogenesis^3^,^4^,^5^. Conventionally, human umbilical vein endothelial cells (HUVECs) and human aortic endothelial cells (HAECs) are representative endothelial cell types isolated from human blood vessels, and both cell types show similar cellular characteristics and morphology^6^. However, the differences in functional characteristics between HUVECs and HAECs have not yet been fully defined.

Considering their different cellular origins, HUVECs and HAECs could have different cellular characteristics and several studies have suggested that endothelial cells have their own transcriptional and phenotypic characteristics depending on origin. For instance, the orphan nuclear receptor COUP-TFII is specifically expressed in the venous endothelium and a mutation in COUP-TFII leads to the activation of arterial surface antigen in veins^7^. Notch ligands and receptors are known to be expressed differently in HUVECs and HAECs^8^.

Angiogenesis-related growth factors such as vascular endothelial growth factor (VEGF) or fibroblast growth factor (FGF) are known as important regulators of angiogenesis. During the *in vivo* vascular sprouting process, VEGF induces the polarization of endothelial cells and contributes to the determination of tip cell formation^9^. Simultaneously, Notch signaling converts adjacent cells to stalk cells, leading to VEGF receptor expression^10^,^11^. FGF has also been reported as involved in angiogenesis through loss-of-function studies. Previous studies suggested that the migratory response induced by FGF2 stimulation was distinct in different endothelial cell types; however, FGF2 represented a mild effect on the major guiding cue^12^. Mice lacking individual FGFs revealed a variety of phenotypes, ranging from early embryonic lethality to mild defects^13^,^14^,^15^, suggesting that FGFs act in a developmental stage-specific manner. In addition, FGF ligands or their unique expression patterns in specific tissues determine the possibility of endothelial cell protrusion. FGF2 deficiency in endothelial cells causes defects in endothelial cell integrity^16^,^17^, and FGF2 enhances endothelial cell proliferation and vessel repair in injured vessels^18^,^19^. FGF5 is well known to have tight connection with hair growth cycle^20^, and gene transfer of FGF5 into injured myocardium was reported to promote blood flow and enhanced vessel formation^21^,^22^. However role of FGF5 for angiogenesis has not been known much. The role of FGF ligands and receptors in different endothelial cell types is also poorly understood.

Recently, three-dimensional (3D) microfluidic angiogenesis systems have been adopted in vascular research^23^,^24^,^25^. They can form 3D tube-like angiogenic structures, perfectly circular and randomly distributed in 3D extracellular matrix (ECM) scaffold. They have advantage of mimicking *in vivo*-like microenvironments of chemical gradients and physical stiffness in the scaffold, and also provide *in situ* quantitative analysis on the angiogenic morphology under various stimuli^26^,^27^,^28^. In this study, the features of the 3D microfluidic angiogenesis system were successfully adopted by *in vivo* mimicking of vascular sprouting via a VEGF-A gradient^29^ and a precise computational simulation^25^ to a detailed comparison of the angiogenic potential of HAECs and HUVECs.

## Results

### HUVECs and HAECs exhibit similar cellular characteristics in a 2D culture system

**Figure 1:**
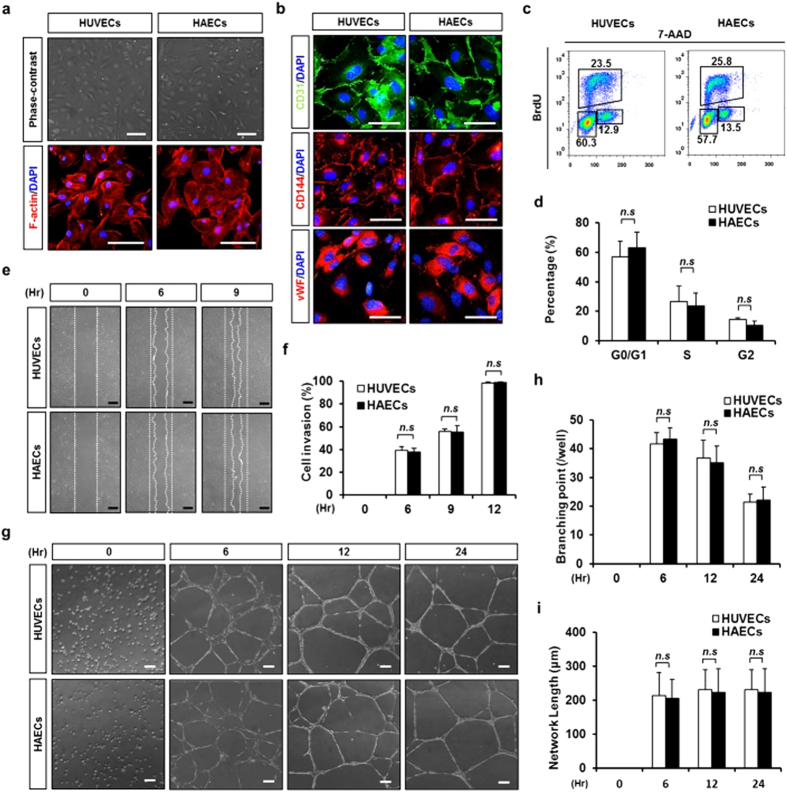
HUVECs and HAECs have similar cellular characteristics in a two-dimensional culture dish. (**a**) Representative phase contrast and F-actin (red) immunofluorescence images of HUVECs and HAECs in a 2D culture dish. Nucleus were stained with DAPI (blue). Scale bar = 100 μm. (**b**) Endothelial cell characterization using immunofluorescence staining with primary antibody followed by CD31 (green), CD144 (red), vWF (red), and DAPI (blue) staining. Scale bar = 50 μm. (**c**) BrdU-incorporated HUVECs and HAECs were measured using flow cytometry. (**d**) Quantification of G0/G1-phase, S-phase or G2-phase cell percentage in HUVECs and HAECs. *n* = 3, *n.s.*; non-significant. (**e**) A phase contrast image of migrated cells observed by the time-dependent wound healing assay. Scale bar = 200 μm. (**f**) Quantification of cell invasion rate against time. *n* = 5. (**g**) Phase contrast images obtained during network formation assay from 0–24 hr. Scale bar = 50 μm. (**h**) Quantification of branching points per visual field. *n* = 4. (**i**) Quantification of network length per visual field. *n* = 4. *n.s.*: non-significant.

We compared the cellular characteristics of HUVECs and HAECs in a 2D culture system. Both cell types showed a similar endothelial cell-specific cobblestone appearance (Fig. 1a). Immunofluorescence images show that CD31, CD144 and vWF were ubiquitously expressed in both cell types (Fig. 1b). Bromodeoxyuridine (BrdU) incorporation rate was also similar between the HUVECs and HAECs (Fig. 1c,d). Results from scratched wound-healing assays also showed similar *in vitro* wound closure rates (Fig. 1e,f). Both HUVECs and HAECs showed a similar *in vitro* network formation, which was maintained up to 72 hours on Matrigel without any morphological differences (Fig. 1g–i and Supplementary Fig. 1).

### HAECs represent stronger angiogenic sprouting into type I collagen than HUVECs in the 3D microfluidic angiogenesis system

**Figure 2:**
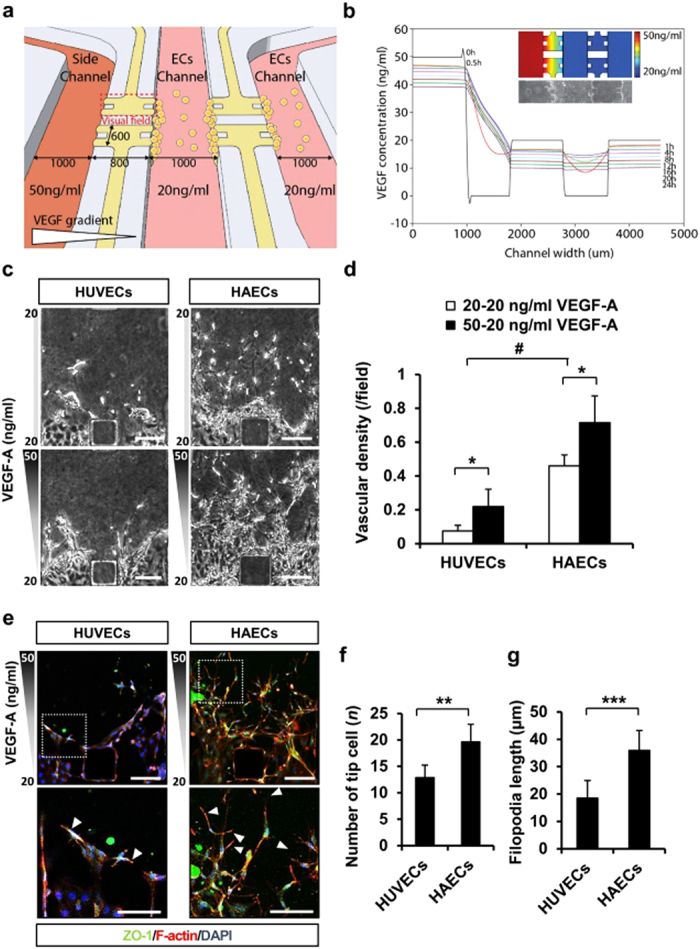
HAECs have more angiogenic potential than HUVECs under VEGF-A stimulation in a 3D microfluidic angiogenesis system. (**a**) Schematic view of the 3D microfluidic angiogenesis system. (**b**) Simulation of the VEGF-A gradient during culture in the 3D microfluidic angiogenesis system. (**c**) Representative phase contrast images of HUVECs and HAECs stimulated with VEGF-A at day 3. Scale bar = 150 μm. (**d**) Measurement of vascular density per field in HUVECs and HAECs. *n* = 6. (**e**) Immunofluorescence images of HUVECs and HAECs in the 3D microfluidic angiogenesis system obtained by incubating the cells with antibody followed by ZO-1 (green), F-actin (red), and DAPI (blue). Scale bar = 150 μm. White arrows indicate different filopodia extensions of tip cells. (**f**) Measurement of tip cell number in HUVECs and HAECs. *n* = 6. (**g**) Measurement of filopodia length (μm) of HUVECs and HAECs (per field). *n* = 6. *p < 0.05, **p < 0.01 and ***p < 0.001 versus HUVECs, and ^*#*^*p* < 0.05 versus 20-20 ng/ml VEGF-A group.

In the 2D culture systems, HUVECs and HAECs showed similar angiogenic appearances, which were verified by 3D microfluidic angiogenesis system. VEGF-A (50 ng/ml) was supplied to the side channel and VEGF-A (20 ng/ml) was added to the two expansion channels (ECs) to generate a VEGF-A gradient in the collagen scaffold. Endothelial cells (2 × 10^6^ cells/ml) were seeded into the two ECs (Fig. 2a). The diffusion profile, estimated using COMSOL, confirmed that the VEGF-A concentration gradually decreased according to the position of all channels (Fig. 2b). In the 3D microfluidic angiogenesis system, the endothelial cells induced new sprouts that invaded into the scaffold in a VEGF-A gradient dependent manner (Fig. 2c,d and Supplementary Fig. 2). Both cell types did not possess angiogenic potential in the absence of VEGF-A; however, the vascular density of HAECs was 6.09-fold higher at 20-20 ng/ml VEGF-A and 3.27-fold higher at 50-20 ng/ml VEGF-A than that of the HUVECs. Lumen formation was observed in both endothelial cells, with very small differences in lumen diameter between HUVECs and HAECs (Supplementary Fig. 3). Interestingly, immunofluorescence staining with ZO-1 and F-actin showed a 1.52-fold higher tip cell number and a 1.95-fold longer filopodia length, respectively, in HAECs relative to HUVECs (Fig. 2e–g). The higher angiogenic potential of HAECs (compared to HUVECs) was maintained under hypoxic conditions of 5% and 10% oxygen. Interestingly angiogenic potential of both cell types were the highest in mild hypoxic condition of 10% oxygen, than the other two conditions of 5% and 20% oxygen (Supplementary Fig. 4).

### Increased HAECs angiogenic sprouting potential was independent of gel stiffness and type

**Figure 3:**
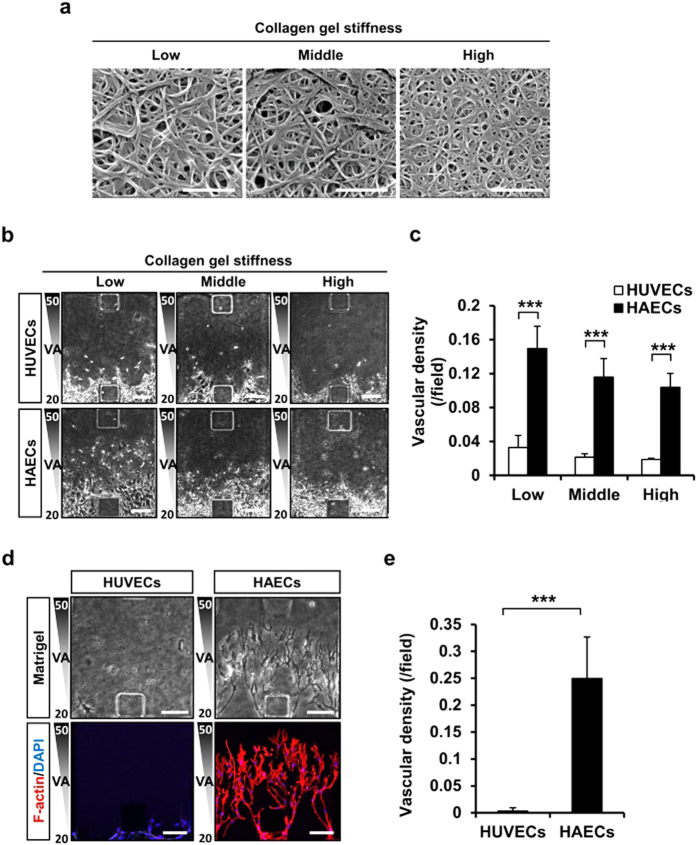
HAECs have greater angiogenic potential than HUVECs owing to differences in collagen fiber stiffness and porosity. (**a**) Electron microscopy images of the effect of pH on collagen fiber stiffness. Scale bar = 1 μm. (**b,c**) Phase contrast images of collagen fiber stiffness in the 3D microfluidic angiogenesis system and quantification of vascular density. Scale bar = 150 μm. (**d,e**) Phase contrast and immunofluorescence images of the vascular density of HUVECs and HAECs filled with laminin-enriched gel obtained by incubating the cells with antibody followed by F-actin (red) and DAPI (blue) staining. Scale bar = 150 μm. *p < 0.05, **p < 0.01 and ***p < 0.001 versus HUVEC group.

The correlation of type I collagen gel stiffness and collagen fiber diameter was assessed by adjusting collagen solution pH during polymerization, confirming that smaller diameter of collagen fiber in higher pH increased gel stiffness^30^. The diameter of individual collagen fiber can be measured by electron microscopy (Fig. 3a). Invasion of tubular structures of both HAECs and HUVECs into the stiff collagen gel was found to be significantly reduced. However, the higher angiogenic potential of HAECs than HUVECs was maintained in both environments with high and low stiffness (Fig. 3b,c). The protruding region area and perimeter of cellular invasion was approximately 2.73-fold and 1.79-fold higher, respectively, in HAECs relative to HUVECs. These results demonstrate that angiogenic potential of HAECs is higher than HUVECs in both stiff and soft type I collagen gel (Supplementary Fig. 5). When the collagen gel was exchanged with the laminin-enriched Matrigel (growth factor reduced), HAECs successfully sprouted into the Matrigel where HUVECs failed (Fig. 3d,e). These data suggest that in the 3D microfluidic angiogenesis system, HAECs have stronger angiogenic potential that is independent of gel stiffness and components.

### FGF2 and FGF5 expression was higher in HAECs than in HUVECs

**Figure 4:**
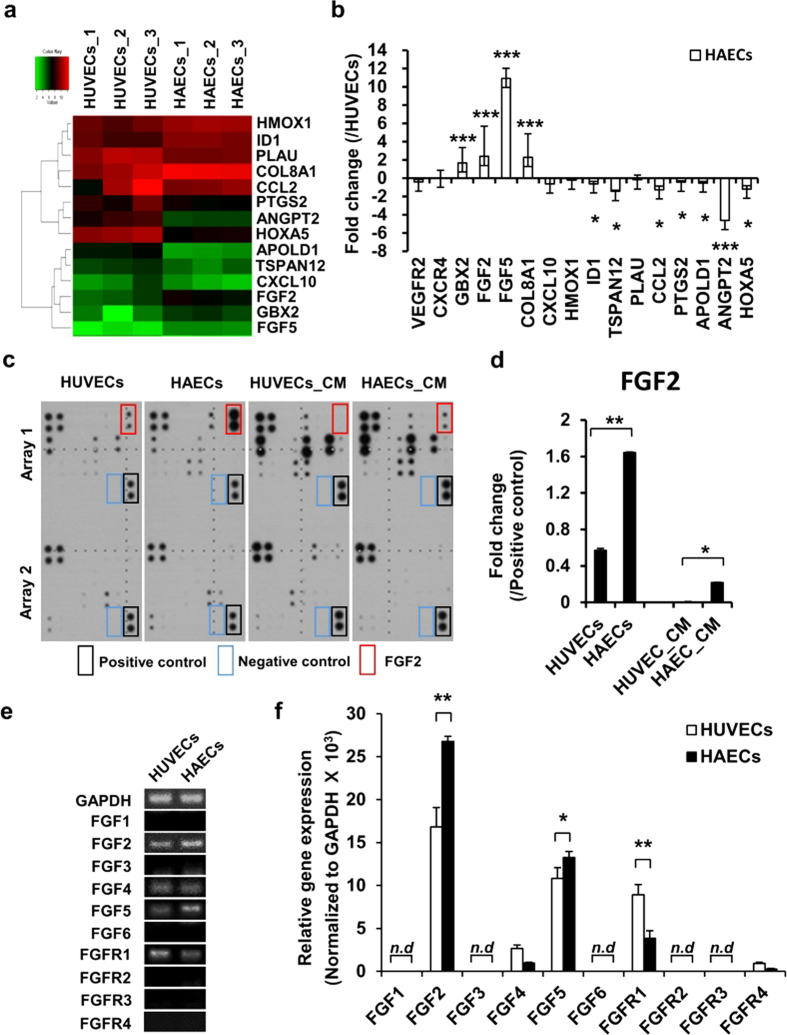
FGF2 and FGF5 are highly expressed in HAECs relative to HUVECs. (**a**) Microarray analysis of the angiogenesis-related mRNA expression level of HUVECs and HAECs gated for two-fold higher expressed genes. *n* = 3. (**b**) Quantitative PCR to confirm the mRNA expression level of HUVECs and HAECs. Values are the average of three independent experiments; they are normalized to the expression levels in HUVECs. *n* = 3. *p < 0.05, **p < 0.01 and ***p < 0.001 versus HUVECs. (**c**) Angiogenesis cytokine array of cell lysates and conditioned medium for both cell types. (**d**) Quantification of FGF2 protein levels in the cell lysate and conditioned medium *n* = 3. *p < 0.05 and **p < 0.01 versus HUVECs group or HUVEC_CM group. (**e**) Determination of endogenous FGF ligand and receptor levels in HUVECs and HAECs using semi-quantitative RT-PCR. (**f**) Quantitative PCR was performed as a confirmatory test. mRNA expression relative to the mean values observed in HUVECs. *n* = 3. *n.d.*: non-detection. *p < 0.05 and **p < 0.01 and versus HUVECs group.

Microarray analysis was performed to identify differentially expressed transcripts in HAECs and HUVECs. HUVECs and HAECs had similar transcriptional expression profiles with 96% accordance (Supplementary Fig. 6a). Factors showing two-fold increase were selected among angiogenesis-related genes using the “GO term” process (Fig. 4a and Supplementary Fig. 6b). The angiogenesis-related genes selected by microarray analysis were verified by quantitative real-time RT-PCR. GBX2 (1.68-fold), FGF2 (2.38-fold), FGF5 (10.92-fold), and COL8A1 (2.27-fold) were upregulated and ID1 (0.62-fold), TSPAN12 (1.46-fold), CCL2 (1.27-fold), PTGS2 (0.44-fold), APOLD1 (0.53-fold), ANGPT2 (4.63-fold), and HOXA5 (1.19-fold) were downregulated in HAECs, which was different from the expression profile in HUVECs (Fig. 4b). FGF2 and FGF5 mRNAs were most highly expressed in HAECs relative to HUVECs. FGF2 protein level was significantly higher in both HAEC lysate (2.88-fold) and HAEC-conditioned medium (61.4-fold) relative to HUVEC, as determined by angiogenesis cytokine arrays (Fig. 4c,d and Supplementary Fig. 7a,b). Semi-quantitative and quantitative real-time RT-PCR of FGF ligands and receptors showed that FGF2, FGF4, FGF5, and FGFR1 were detected in both HAECs and HUVECs; however, FGF2 and FGF5 expression was 1.59-fold and 1.23-fold higher, respectively, and FGFR1 expression was 2.31-fold lower in HAECs relative to HUVECs (Fig. 4e,f).

### FGF2 and FGF5 play a crucial role in HAECs sprouting pattern

**Figure 5:**
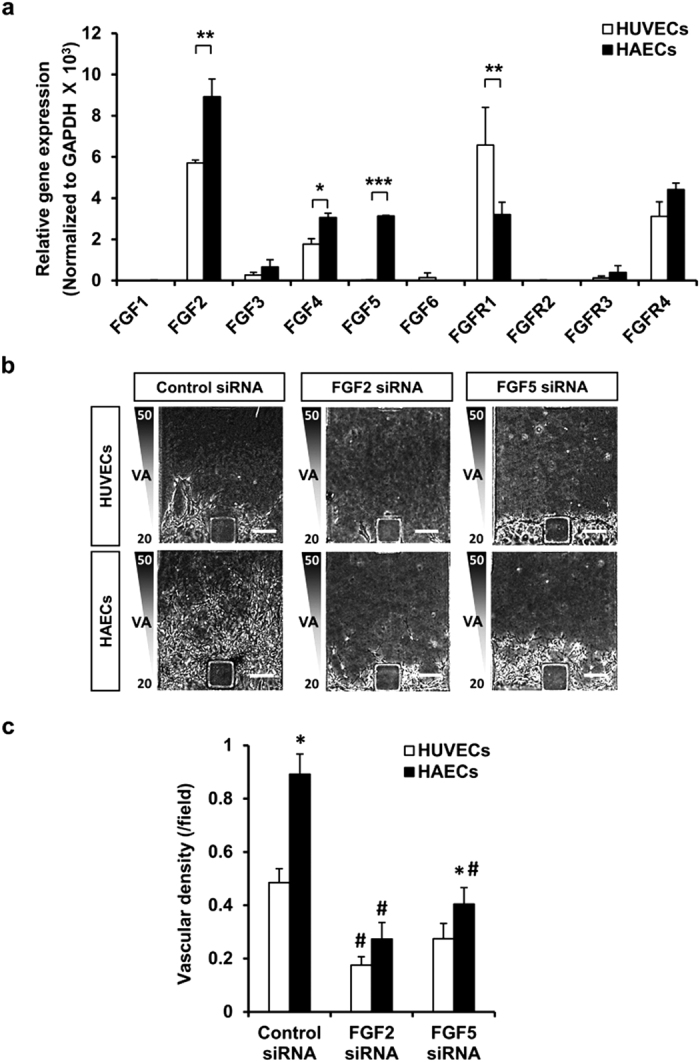
FGF2 and FGF5 are crucial factors for angiogenesis in the 3D microfluidic angiogenesis system. (**a**) Quantitative PCR analysis was performed to confirm mRNA expression levels. mRNA expression relative to the mean values of GAPDH. *n* = 3. *p < 0.05, **p < 0.01 and ***p < 0.001 versus HUVECs group. (**b,c**) siRNA-mediated knockdown of FGF2 and FGF5 in HUVECs and HAECs. Scale bar = 150 μm. *n* = 5. *p < 0.05 versus HUVECs, and ^*#*^*p* < 0.05 versus control siRNA group.

Next, we compared the mRNA expression levels of FGF ligands and receptors in HAECs and HUVECs during the *in vitro* sprouting and invasion processes in the 3D microfluidic angiogenesis system. Quantitative real-time RT-PCR showed that FGF2 (1.56-fold) and FGF5 (110.15-fold) were strikingly upregulated in HAECs relative to HUVECs in the 3D microfluidic angiogenesis system (Fig. 5a). Furthermore, we observed that FGF2 recombinant protein treated endothelial cells significantly increased vascular density; however, when treated with the FGFR inhibitor SU5402, HAECs sprouting with elongated morphology and prominent filopodia were impaired (Supplementary Fig. 8a–d). Finally, we compared the effect of FGF2 and FGF5 on HAECs and HUVECs sprouting angiogenic potential using FGF2 small interfering RNA (siRNA) or FGF5 siRNA in the 3D microfluidic angiogenesis system. Transient transfection of FGF2 siRNA dramatically abolished the vascular invasion of both HUVECs and HAECs into ECM scaffold; interestingly, FGF5 siRNA transfection significantly reduced the angiogenic sprouting of HAECs over that of HUVECs (Fig. 5b,c). Expression of phospho-fibroblast growth factor receptor 1 (p-FGFR1) increased in HAECs rather than HUVECs when treated with recombinant FGF2 and FGF5 (Supplementary Fig. 9a–c). Intrinsic FGF2 and FGF5 in HAECs seemed to increase angiogenic potential via FGFR phosphorylation.

## Discussion

The study presents that; (i) HAECs show higher angiogenic potential than HUVECs under VEGF-A stimulation only in 3D microfluidic angiogenesis system, and (ii) although endogenous FGF2 and FGF5 expression in both cell types is a crucial regulator of angiogenesis, increased expression of FGF5 rather than that of FGF2 extends greater angiogenic potential to HAECs.

The 3D microfluidic angiogenesis system used in the study is found to be powerful for understanding stereoscopic cellular morphogenesis of 3D cooperative migration and morphogenesis of endothelial cells^31^,^32^. For example, the regulation of type 1 collagen solution pH during gelation is known to alter the gel stiffness^33^ in cross-linking of the fibers^34^. In accordance with the previous report, the collagen stiffness was observed to influence angiogenic potential of both HUVECs and HAECs (Fig. 3b,c). ECM-related physical interaction might be a regulator of angiogenic potential. When endothelial cells reside in a complex 3D architecture, they perceive cues from the ECM through cell-cell or chemo-mechanical coupling, and finally generate angiogenic responses^35^. In the present study, both cell types have similar cellular characteristics and function in 2D culture systems; however, HAECs demonstrated significantly higher angiogenic potential than HUVECs into the type I collagen gel in the 3D microfluidic angiogenesis system. This suggests that an intrinsic factor in both cell types might play a role in angiogenic process.

Some angiogenesis-related factors were known to show different expression patterns in each endothelial cell type. The VEGF-VEGFR signaling pathway is known to regulate vessel formation for both arterial and venous endothelial cells^36^,^37^. Moreover, F11R protein, known as a crucial indicator for atherosclerosis, was induced in both HUVECs and HAECs after treatment with the same concentration of TNFα and INFβ^38^. On the other hand, VCAM-1 expression in HAECs was significantly increased by treatment with a 20-fold higher concentration of sCD40L than that used in HUVECs^39^. EphrinB2 was also highly expressed in HAECs, whereas EphB4 was only expressed in HUVECs but not in HAECs^40^. In addition, hypoxia potentiated agonist evoked responses in arterial endothelial cells but not in venous endothelial cells^41^. These data suggest that several angiogenesis-related factors show different expression patterns in each endothelial cell type. Microarray experiments in the present study observed that GBX2 and COL8A were highly expressed in HAECs, but ID1, TSPAN12, CCL2, PTGS2, APOLD1, ANGPT2, and HOXA5 were highly expressed in HUVECs. They had a good correlation with the previous studies. In different types of endothelial cells, pharyngeal arch artery development was involved in the upregulation of GBX2^42^. COL8A was also reported to be associated with VEGF-A in angiogenesis-dependent macular degeneration^43^, whereas ANGPT2, a regulatory factor for vessel remodeling^44^, was highly expressed in HUVECs relative to HAECs. These results suggest that the angiogenic potential of HUVECs and HAECs are influenced by different growth factors and cytokines, and they depend on cell origin. In addition, these results suggest that GBX2, COL8A, and ANGPT2 may be crucial factors for determining the diversity of specialized endothelial cell types.

Cytokines and growth factors secreted from endothelial cells also determine endothelial cell characteristics in an autocrine manner. In the angiogenesis cytokine array performed on cell lysates, EGF, FGF2, uPAR, TIMP1, and TIMP2 are highly expressed in HAEC lysates relative to HUVEC lysates. Interestingly, FGF2 is only expressed in HAEC-conditioned medium, but not in HUVEC-conditioned medium, suggesting that FGF2 might play an important role in arterial endothelial cell function. In contrast, IL-8 and MMP are highly expressed in HUVECs and ANGPT2 and MCP-1 are highly expressed in both HUVEC lysates and HUVEC-conditioned medium, indicating that these molecules could play a role in venous endothelial cell specification.

FGFs are small polypeptide growth factors that contain signal peptides for secretion to the extracellular environment to promote endothelial cell growth and movement^45^. Four FGF receptors and 22 FGF ligand members have already been reported^46^. FGF signaling has been implicated in many physiological and pathological processes in angiogenesis. VEGF in vertebrate endothelial cells is a fundamental regulator of vasculogenesis, angiogenesis, and lymphangiogenesis in conjunction with multiple other growth factors such as PDGF-BB, TGF-β1, FGF2, S1P, ANGPT1, and ANGPT2, as well as the signaling pathways involving NOTCH and Ephrin^28^. One of the most interesting things about VEGF is that it acts as a critical morphogen connected by FGF2 and primarily acts as a mitogen^47^. Consistent with a previous report, we found the significance of FGF2 and FGF5 during angiogenesis between HUVECs and HAECs under a VEGF-A gradient, especially when the mRNA expression of FGF5 was excessively increased in HAECs. This phenomenon was verified in the 3D microfluidic angiogenesis system. Although the absolute expression level of FGF2 was higher than that of FGF5, the relative fold-change value of FGF5 was higher than that of FGF2 in HAECs. Therefore, FGF5 expression suggests a target as a sprouted endothelial cell marker.

To validate the angiogenic effect of FGF2 and FGF5, we performed various functional assays such as the siRNA technique and FGF inhibitor assessments. First, we confirmed whether FGF2 and FGF5 siRNA could inhibit the formation of sprouted structures. The formation of HAECs vessel-like structures in 3D microfluidic angiogenesis system was significantly inhibited by FGF2 and FGF5 siRNA. Our study demonstrates that the simultaneous treatment of HAECs with FGF2 or FGF5 siRNA impaired cell sprouting from a polygonal to an elongated morphology with prominent filopodia; however, the vascular density inhibited by FGF5 siRNA was not significant in HUVECs. FGF signaling inhibitors such as SU5402 have been effectively used for the functional inhibition of FGFR^48^. Similarly, we observed that SU5402 impaired cell sprouting from a polygonal to an elongated morphology with prominent filopodia (Supplementary Fig. 8c,d). Similarly, we observed that SU5402 impaired cell sprouting from a polygonal to an elongated morphology with prominent filopodia (Supplementary Fig. 8c,d). The previous report suggested that FGF2 mainly regulated angiogenesis at development stage and vascular integrity^49^. In this study, the FGF2 was found to be crucial for angiogenesis in both HAECs and HUVECs, but FGF5 was involved in angiogenesis and vessel patterning only in HAECs under 3D microenvironment. Our understanding of the angiogenesis mechanism, where FGF2 and FGF5 expression contributes to angiogenesis, is still limited. Although a direct correlation among VEGF-A, FGF2, and FGF5 were not fully addressed, but both FGF2 and FGF5 were found to be critical to induce angiogenesis in the presence of VEGF-A.

In conclusion, the study provides evidence that FGF2 and FGF5 are strongly and selectively expressed in HAECs than HUVECs. The 3D microfluidic angiogenesis system helped to apparently characterize morphological features in angiogenic procedure of the endothelial cells, which could not be differentiated in previous 2D assays. HAECs were found to have higher angiogenic potential through the upregulation of FGF2 and FGF5, which could be a therapeutic targets for anti-angiogenic strategy.

## Methods

### Endothelial cell culture

HUVECs (BioBud Inc. Seoul, Korea) and HAECs (LONZA Walkersville Inc. Basel, Switzerland) were cultured according to the manufacturer’s instructions. The culture medium was composed of EGM-2 MV (Contained with 20 ng/ml VEGF-A, insulin growth factor, epidermal growth factor, fibroblast growth factor 2, hydrocortisone, ascorbic acid and RA-1000) supplemented with 5% fetal bovine serum, 100 U/ml penicillin, and 50 U/ml streptomycin in a fully humidified atmosphere of 5% CO_2_ at 37 °C. The medium was changed every two days.

### 3D microfluidic angiogenesis system

Polydimethylsiloxane (Sylgard 184, Dow Chemicals)-based 3D microfluidic angiogenesis systems were used as previously described^25^. Briefly, polydimethylsiloxane chips were bonded with a coverslip by plasma treatment (Femto Science) and immediately coated with poly-L-lysine (P8920, Sigma) solution, followed by incubation at 37 °C for 4 hrs. After coating, the chip was washed and dried at 80 °C for 24 hrs. Type 1 collagen gel (354236, BD Bioscience) was prepared at the desired pH and concentration, injected into the gel, and incubated at 37 °C for 30 min for gelation. After gelation, the channels were filled with growth medium and the cell channels were filled with the cell suspension (1.2 × 10^5^ cells/channel). After seeding, all chips were incubated at 37 °C in a fully humidified atmosphere of 5% CO_2_, and the medium was replaced daily. Human recombinant FGF2 (AF-233, R&D Systems) and SU5402 (572630, Calbiochem) were added into the upper EC channel. Cell migration was monitored daily and images were captured using MetaMorph software (Molecular Devices). Vascular density, capillary forming region perimeter and Capillary forming region area were analyzed with ImageJ software (NIH Image, Bethesda, MD). Vascular density = (Total gel area − Day n gel area)/Total gel area.

### VEGF-A simulation in the 3D microfluidic angiogenesis system

Angiogenic molecules supplied in the 3D microfluidic angiogenesis system were simulated as previously described^25^. The VEGF-A (100-20, PeproTech) gradient and the sprouting angiogenic potential of endothelial cells under a 50-20-20 ng/ml VEGF-A gradient over 24 h were estimated using the finite element method-based computational fluid dynamics code in COMSOL Multiphysics 4.0 (COMSOL Inc.). Diffusion of VEGF-A was simulated using Fick’s second law: (∂C)/(∂t) + r3(−Drc) = 0, where “C” is VEGF-A concentration (mol/m^3^), and “D” is the diffusion coefficient (m^2^/s). VEGF-A concentration was determined subject to the initial conditions of passive VEGF-A supply (all channels were filled with the control medium; 20-20-20 ng/ml VEGF-A containing EGM-2 MV) or active VEGF-A supply (a high concentration was applied to the left channel; 50-20-20 ng/ml VEGF-A containing EGM-2 MV).

### Immunofluorescence staining

For fluorescent staining, cells were fixed with 4% paraformaldehyde (P6148, Sigma) and blocked with 4% bovine serum albumin (A9418, Sigma) in phosphate-buffered saline containing 0.1% Triton X-100 (PBST; X-100, Sigma) at room temperature for 1 hr. The cells were incubated overnight in a humid chamber at 4 °C with primary antibodies: anti-human CD31 antibody (M082301, DAKO) and anti-human ZO-1 antibody (40-2300, Invitrogen), anti-human vWF antibody (A0082, DAKO), and anti-human CD144 (555661, BD). The cells were washed with PBST three times, followed by 1 hr incubation with secondary antibodies: Alexa Fluor 488 goat anti-rabbit IgG (A11008, Invitrogen), Alexa Fluor 594 goat anti-rabbit IgG (A11012, Invitrogen), Alexa Fluor 594 goat anti-mouse IgG (A11005, Invitrogen) and Rhodamine Phalloidin antibody (R415, Invitrogen). After staining the nucleus with 4′,6-diamidino-2-phenylindole (DAPI; D1306, Invitrogen), the cells were mounted with a fluorescent mounting medium (S3023, DAKO). Immunohistochemistry images were acquired using fluorescence microscope (BX61, Olympus) and Zeiss LSM700 confocal fluorescence microscope (Carl Zeiss). Immunofluorescence staining images in 3D microfluidic angiogenesis system were presented by merging of z-stack confocal images.

### Cell proliferation assay

Cell proliferation was analyzed using the fluorescence-activated cell sorting (FACS) BrdU flow kit (559619, BD Bioscience) according to the manufacturer’s instructions. Briefly, BrdU was added to cells and incubated in a CO_2_ incubator at 37 °C for 1 h. The cells were fixed and permeabilized with a buffer (554722, BD Bioscience) for 20 min. After incubation with 30 μg of DNase at 37 °C for 1 h, the cells were washed twice and stained with fluorescein isothiocyanate (FITC)-conjugated anti-BrdU antibody at room temperature for 20 min. After resuspension with 7-AAD-containing buffer, the cells were finally analyzed using flow cytometry (FACS Calibur, BD Bioscience).

### Scratch wound-healing assay

HUVECs and HAECs were seeded into 6-well plates at a density of 4 × 10^5^ cells per well. After 24 h of incubation, confluent cell monolayers were scratched across the midline using a pipette tip, and then scanned at 0, 6, 9, and 12 h using an optical microscope (Optika).

### *In vitro* network formation assay

With about 250 μl of growth factor reduced BD Matrigel (356231, BD Bioscience) was evenly spread on a 24-well culture plate and allowed to solidify at 37 °C. HUVECs and HAECs were spread on a Matrigel-treated wells at a density of 4 × 10^4^ cells per well. The cells were observed microscopically (Optika) and recorded for up to 120 hrs. Phase contrast images were obtained using an Optika visual program. Network length and branching point were quantified with ImageJ software (NIH Image, Bethesda, MD)^50^. Branching point indicates where network meets more than three. Network length was measured from one branching point to the end of another branching point.

### Functional comparison of HUVECs and HAECs regulated by oxygen tension

The fabrication of the 3D microfluidic angiogenesis system for cellular experiment was performed by monitoring the invasion of HUVECs and HAECs under normoxia and hypoxia. Both of endothelial cells were harvested by trypsinization and seeded middle and under channel in 3D microfluidic angiogenesis system to a final cell density of 1.2 × 10^5^ cells/channel containing 50-20-20 ng/ml VEGF gradient. 21% O_2_, 5% CO_2_ and 74% N_2_ was supplied incubator to create normoxic condition. Hypoxia was then generated by switching O_2_ gas mixture from 21% to 10% or 5%. 10% O_2_, 5% CO_2_ and 85% N_2_ was supplied incubator to create mild hypoxic condition. In addition, 5% O_2_, 5% CO_2_ and 90% N_2_ was supplied incubator to create harsh hypoxic condition. Phase contrast images were acquired at day 3 after plating on 3D microfluidic angiogenesis system.

### Semi-quantitative and quantitative real-time RT-PCR

Total RNA was extracted from HUVECs and HAECs using Trizol reagent (TR-118, MRC) according to the manufacturer’s recommendations. About 500 ng of RNA was reverse-transcribed into complementary DNA (cDNA) using M-MLV reverse transcriptase (28025-013, Invitrogen). Polymerase chain reaction using iQ^TM^ SYBR Green supermix (170-8880, Bio-Rad) and the indicated primers was performed using the AB PCR system (Applied Biosystems) or MYiQ2 detection system (Bio-Rad). GAPDH (Forward: 5′-ACCACCATGGAGAAGGC-3′, Reverse: 5′-GGCATGGACTGTGGTCATGA-3′) was used as an internal standard. Primer sequences and product sizes are presented in Supplemental Table 1.

### Microarray gene expression profile

For microarray analysis, total RNA was extracted using the RNeasy Plus Mini kit (74134, Qiagen). Microarray fabrication was conducted at the BML Corporation using their proprietary technology. Fragmented RNA was hybridized on a HumanHT-12 v4 Expression BeadChip (BD-103-0204, Illumina) by incubating at 58 °C for 16 h. The hybridization mixture was detected with Cy3-Streptavidin (PA431001, GE Healthcare). The chips were washed, dried, and scanned using the Bead Array Reader (Illumina) and raw data were obtained using GenomeStudio software V2011.1 (Illumina). All genes representing ≥2-fold difference between the two groups were selected.

### Angiogenesis cytokine array

The experiments were performed according to the RayBio® Human Angiogenesis Antibody Array C series 1000 (AAH-ANG-1000, RayBiotech) guidelines. Briefly, array A and B membranes were incubated with blocking buffer for 30 min. After removing the blocking buffer, the membranes were incubated overnight with cell lysates in a humid chamber at 4 °C. The membranes were washed two times and incubated with the antibody cocktail for 2 h. After the addition of HRP-conjugated streptavidin antibody for 2 h, the membranes were exposed to X-ray film using the detection buffers C and D. Intensity of each blot spot was quantified using Quantity One software (Bio-Rad). The data were normalized using positive and negative controls.

### Small interfering RNA assay

About 20 nM FGF2 siRNA, FGF5 siRNA, and AccuTarget negative control siRNA (SN-1003, Bioneer) were transfected using lipofectamine 2000 transfection reagent (11668-019, Invitrogen). The transfected cells were incubated for 6 h in a transfection reagent mixture and maintained for 24 h in EGM-2 MV. After transfer to the 3D microfluidic angiogenesis system, the cells were incubated for 48 h and then harvested for the measurement of FGF2 and FGF5 mRNA. The FGF2 siRNA antisense sequence was as follows: 5′-UAUACUGCCCAGUUCGUUUCAGUGC-3′. FGF5 siRNA (M-011972-01-0005, Dharmacon) target sequences were as follows: 5′-CAUAAGUUGUAUAGGCUAA-3′, 5′-CAACAAUAAGCCACGUCAA-3′, 5′-GCAAGUUCAGGGAGCGUUU-3′, and 5′-GUAUUGAAGUCACGUCAUU-3′.

### Western blot

HUVECs and HAECs were washed twice with PBS, and lysis with 1 mM phenylmethylsulfonyl fluoride (P7626, Sigma) contained 1X cell lysis buffer (9803, Cell signaling). Quantitative analysis of samples were determined with a Bradford assay dye reagent (500-0006, Bio-Rad). Fifteen μg of sample protein was mixed with 1X loading dye and boiled for 5 min, and electrophoresis on 10% SDS-polyacrylamediume gel. After transferred to the polyvinylidene fluoride membrane (ISEQ00010, Millipore), membranes were blocked in 5% skimmilk contained 1X TBST (WH400028806, 3M) for 1 hr. The membranes were incubated with anti-p-FGFR1 (1:1000, ab173305, abcam), anti-FGFR1 (1:1000, 9740, cell signaling) and anti-GAPDH (1:10000, G8795, Sigma) antibody at 4 °C for overnight. Next, the membranes were washed three times in TBST and incubated with a horseradish peroxidase-conjugated secondary anti-mouse HRP antibody (1:7000, SC-2005, Santa Cruz Biotechnology) and anti-rabbit HRP antibody (1:7000, SC-2030, Santa Cruz Biotechnology) in TBST at room temperature for 90 min. Chemiluminescence were visualized using Amersham ECL Prime Westerm Blotting Reagent (RPN2232SK, GE Healthcare Life Sciences) and exposed to X-ray film. Quantification of blotting intensity were performed using Quantity One (Bio-Rad) program.

### Statistics

Data values are presented as the mean ± standard deviation (SD). Statistical significance of the mean values was confirmed by Student’s t-test or ANOVA followed by Student-Newman-Keuls test. *P* < 0.05 indicates statistical significance. All statistical analysis were performed using SigmaStat3.5 (SPSS, Chicago, IL).

## Additional Information

**How to cite this article**: Seo, H.-R. *et al*. Intrinsic FGF2 and FGF5 promotes angiogenesis of human aortic endothelial cells in 3D microfluidic angiogenesis system. *Sci. Rep.* **6**, 28832; doi: 10.1038/srep28832 (2016).

## Supplementary Material

Supplementary Information
