# Supplementary material for: Intrinsic FGF2 and FGF5 promotes angiogenesis of human aortic endothelial cells in 3D microfluidic angiogenesis system
Source: Sci Rep. 2016 Jun 30;6:28832. doi: 10.1038/srep28832 (PMC4928073; doi:10.1038/srep28832)
Supplement: Supplementary Information [file srep28832-s1.pdf]

Supplementary Information

Intrinsic FGF2 and FGF5 promotes angiogenesis of human aortic endothelial cells in 3D microfluidic angiogenesis system

Ha-Rim Seo[1,*], Hyo Eun Jeong[2,*], Hyung Joon Joo[1], Seung-Cheol Choi[1], Chi-Yeon Park[1], Jong-Ho Kim[1], Ji-Hyun Choi[1], Long-Hui Cui[1], Soon Jun Hong[1], Seok Chung[2,†] & Do-Sun Lim[1,†]

[1]Department of Cardiology, Cardiovascular Center, College of Medicine, Korea University, 126-1, 5-ga, Anam-dong, Seongbuk-gu, Seoul 136-705, Republic of Korea.

[2]Department of Mechanical Engineering, #512B, Innovation Hall, Korea University, 5-ga, Anam-dong, Seongbuk-gu, Seoul 136-701, Republic of Korea.

[*] These authors equally contributed to this work

[†] These authors equally supervised this work

Address correspondence to:

* Do-Sun Lim

Department of Cardiology, Cardiovascular Center, College of Medicine, Korea University, 126-1, 5-ga, Anam-dong, Seongbuk-gu, Seoul 136-705, Republic of Korea

Phone: +82-2-920-5445; Fax: +82-2-927-1478; E-mail: dslmd@kumc.or.kr

* Seok Chung

Department of Mechanical Engineering, #512B, Innovation Hall, Korea University, 5-ga, Anam-dong, Seongbuk-gu, Seoul 136-701, Republic of Korea

Phone: +82-2-3290-3352; Fax: +82-2-926-9290; E-mail: sidchung@korea.ac.kr

Supplementary Figure 1

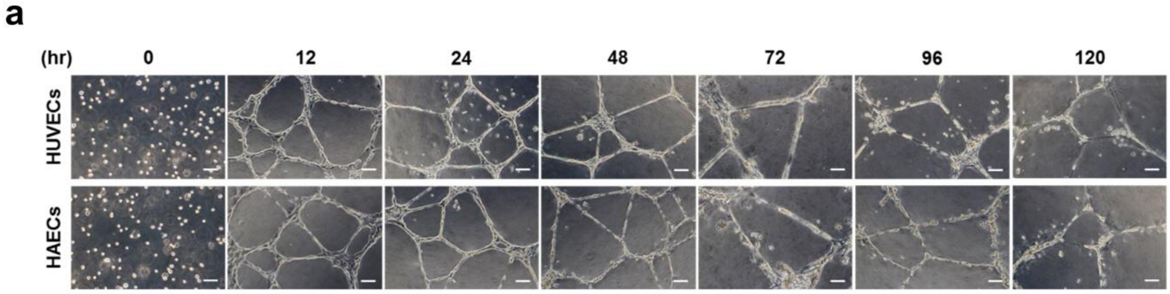

Supplementary Figure 1. *In vitro* Matrigel tube forming assay up to 120 hrs. (**a**) Phase contrast images obtained during network formation assay from 0–120 hrs between HUVECs and HAECs. Scale bar = 100 μm.

Supplementary Figure 2

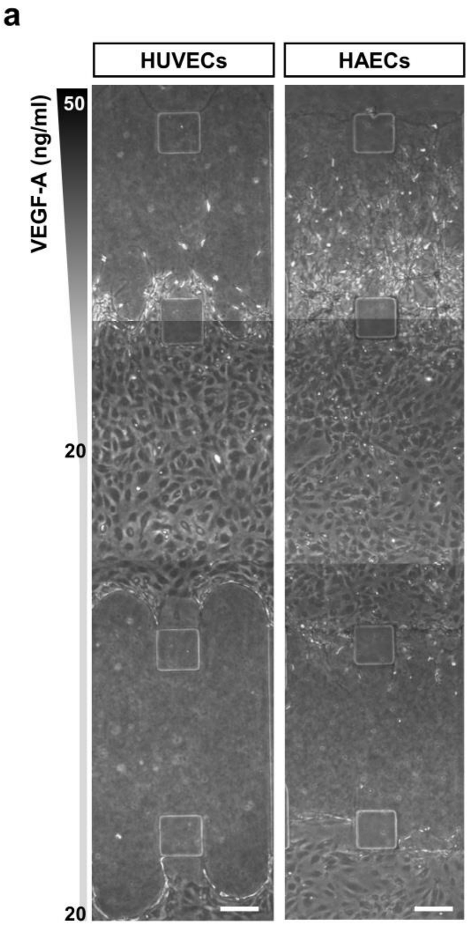

Supplementary Figure 2. Different sprouting patterns of HUVECs and HAECs in the 3D microfluidic angiogenesis system. (**a**) Phage contrast images of whole 3D microfluidic angiogenesis system channels under VEGF-A stimulation. Phase contrast image of the 3D microfluidic device culture stimulated with a VEGF-A gradient of HUVECs and HAECs. Scale bar = 150 μm.

Supplementary Figure 3

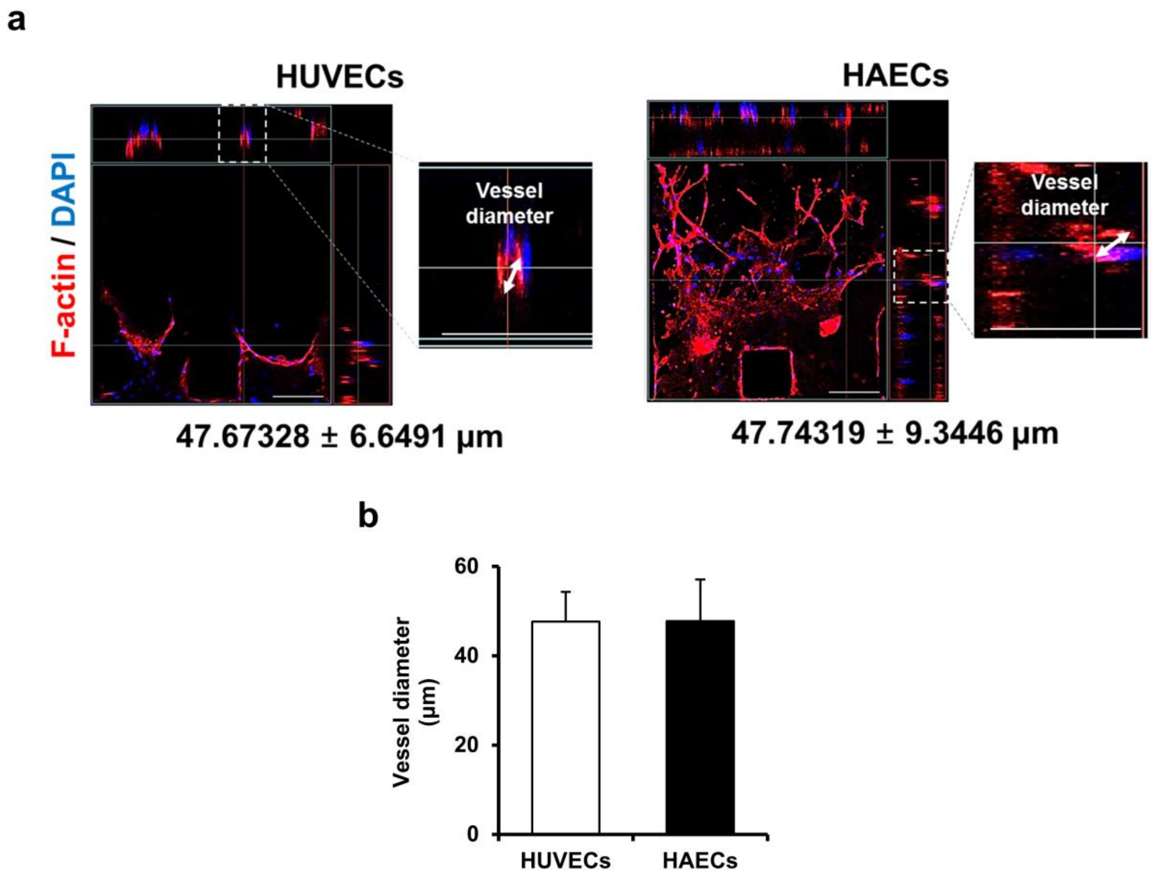

Supplementary Figure 3. Measurement of vessel diameter in HUVECs and HAECs. (**a**) Immunofluorescence staining with F-actin (red) and orthographic projection derived from a z-stack of HUVECs and HAECs. Nucleus were stained by DAPI (blue). Scale bar = 150 μm. (**b**) Quantification of vessel diameter between HUVECs and HAECs. n = 10.

Supplementary Figure 4

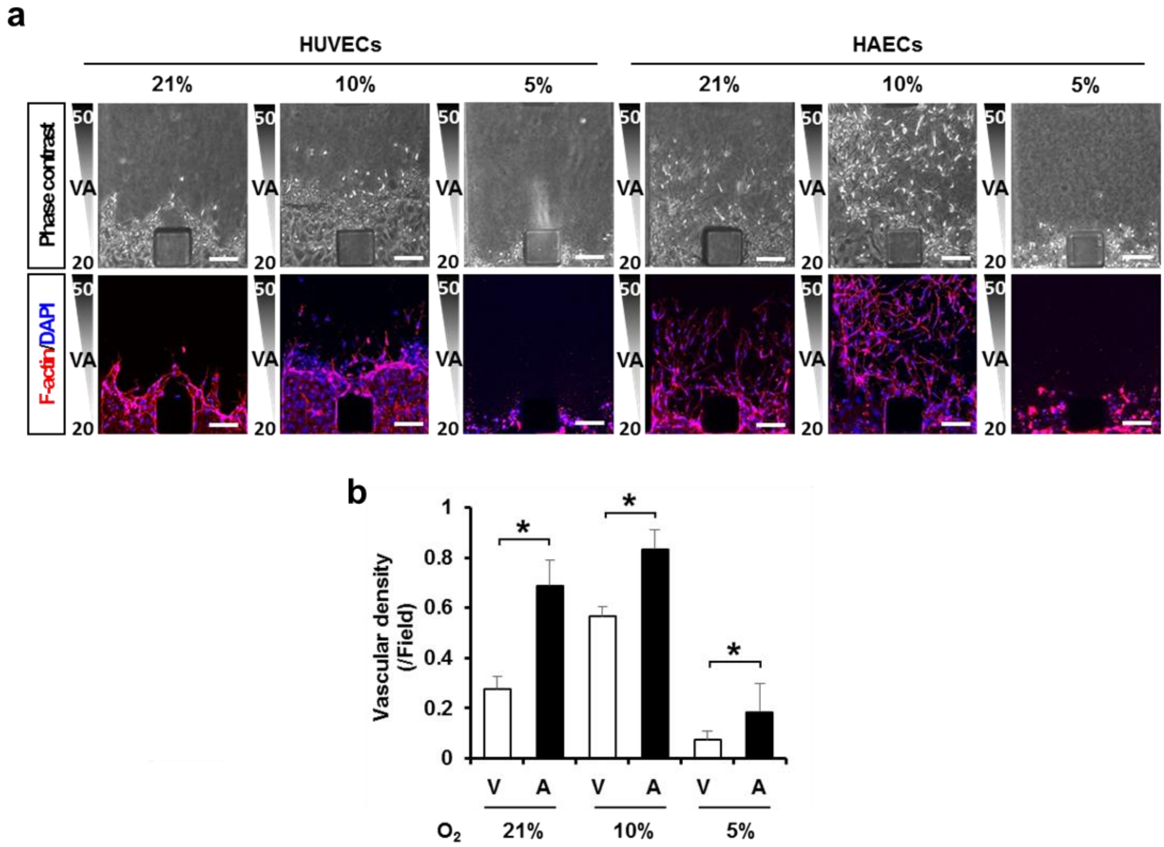

Supplementary Figure 4. Effect of hypoxic condition between HUVECs and HAECs in 3D microfluidic angiogenesis system. (**a**) Phase contrast image of HUVECs and HAECs in the 3D microfluidic angiogenesis system with different oxygen concentration (contained 21%, 10%, and 5% O2). Immunofluorescence staining using antibody followed by F-actin (red) and DAPI (blue). Scale bar = 150 µm. (**b**) Quantification of the rate of vascular density per field in different oxygen concentration between HUVECs and HAECs. 'V' was indicated HUVECs and 'A' was indicated HAECs. $n = 6$. $^{*}p < 0.05$ versus HUVECs group.

Supplementary Figure 5

a

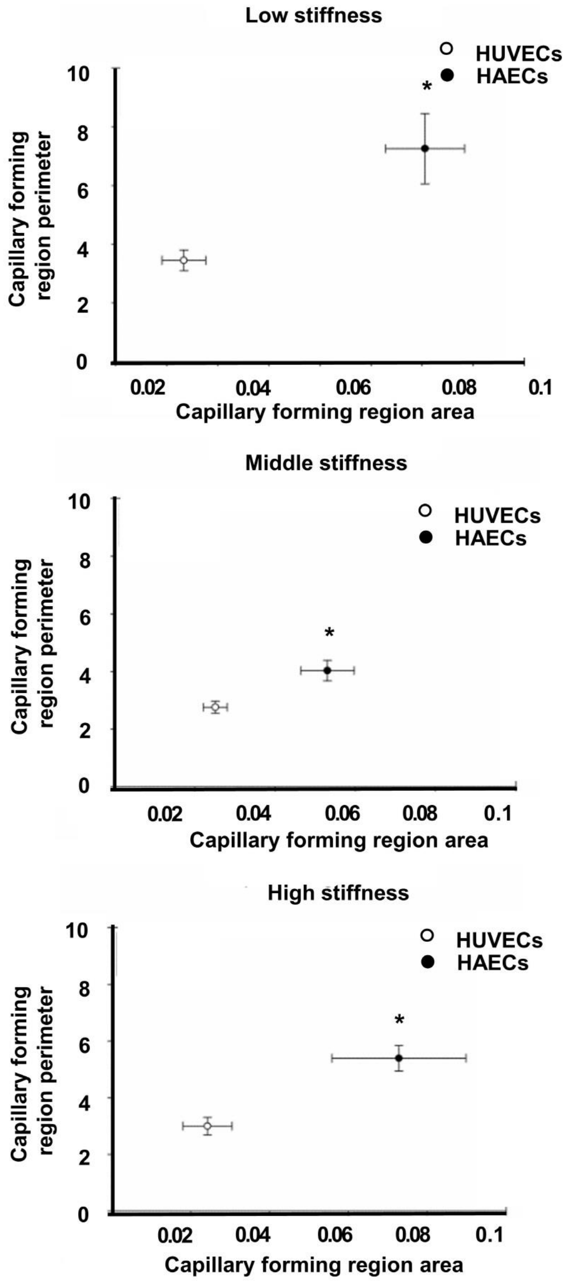

Supplementary Figure 5. Differences in sprouting pattern and other parameters between HUVECs and HAECs according to collagen gel stiffness. (**a**) Comparison of the perimeter and area of the capillary-forming region in HUVECs and HAECs. $n = 4$. $^{*}p < 0.05$ *versus* HUVECs group.

Supplementary Figure 6

a

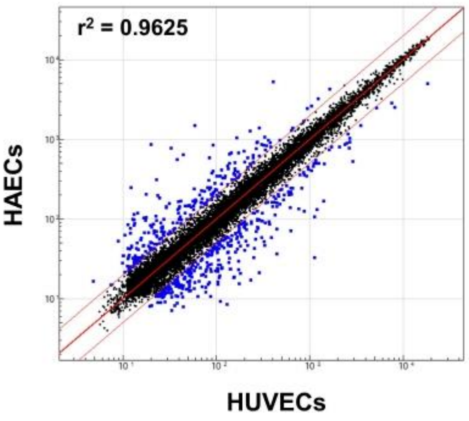

b

Gene	NCBI Accession Number	Fold Change (log 2)
GBX2	NM_001485.2	1.85508307
FGF2	NM_002006.3	1.83315735
FGF5	NM_004464.3	1.66399121
COL8A1	NM_020351.2	1.42960853
CXCL10	NM_001565.1	1.28796940
HMOX1	NM_002133.1	1.12995061
ID1	NM_181353.1	1.07554140
TSPAN12	NM_012338.3	-1.01980168
PLAU	NM_002658.2	-1.06941479
CCL2	NM_002982.3	-1.33719416
PTGS2	NM_000963.1	-1.58895890
APOLD1	NM_030817.1	-2.73906490
ANGPT2	NM_001118888.1	-2.78391438
HOXA5	NM_019102.2	-3.01344519

Supplementary Figure 6. mRNA expression scatter plot between HUVECs and HAECs. (**a**) mRNA expression correlation between HUVECs and HAECs. (**b**) List of typical angiogenesis-related factors sorted according to the "GO-term" document profile.

Supplementary Figure 7

a

Array 1

POS	POS	NEG	NEG	Angiogenin	EGF	ENA-78	FGF2
POS	POS	NEG	NEG	Angiogenin	EGF	ENA-78	FGF2
GRO	IFN-γ	IGF-1	IL-6	IL-8	LEPTIN	MCP-1	PDGF-BB
GRO	IFN-γ	IGF-1	IL-6	IL-8	LEPTIN	MCP-1	PDGF-BB
PIGF	RANTES	TGF-β1	TIMP-1	TIMP-2	Thrombopoietin	VEGF	VEGF-D
PIGF	RANTES	TGF-β1	TIMP-1	TIMP-2	Thrombopoietin	VEGF	VEGF-D
						NEG	POS
						NEG	POS

Array 2

POS	POS	NEG	NEG	Angiopoietin-1	Angiopoietin-2	Angiostatin	Endostatin
POS	POS	NEG	NEG	Angiopoietin-1	Angiopoietin-2	Angiostatin	Endostatin
G-CSF	GM-CSF	I-309	IL-10	IL-1α	IL-1β	IL-2	IL-4
G-CSF	GM-CSF	I-309	IL-10	IL-1α	IL-1β	IL-2	IL-4
I-TAC	MCP-3	MCP-4	MMP-1	MMP-9	PECAM-1	Tie-2	TNF-α
I-TAC	MCP-3	MCP-4	MMP-1	MMP-9	PECAM-1	Tie-2	TNF-α
uPAR	VEGFR2	VEGFR3					POS
uPAR	VEGFR2	VEGFR3					POS

b

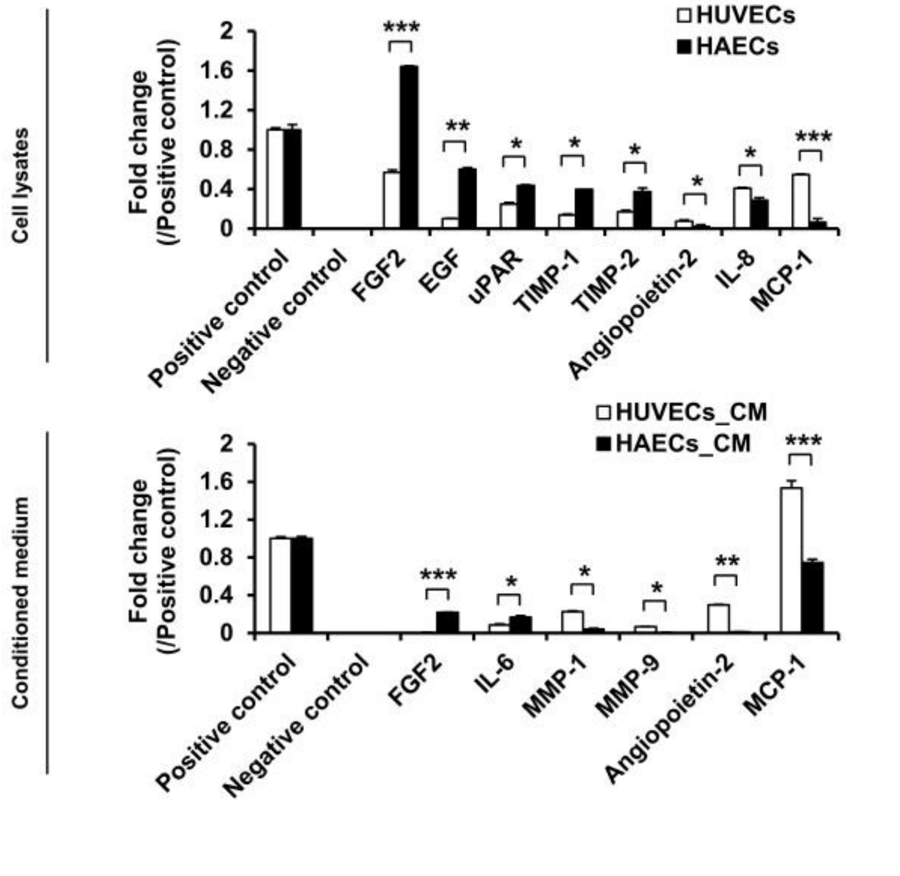

Supplementary Figure 7. Quantification of various protein levels between HUVEC or HAEC lysates and conditioned media. (**a**) Template showing the location of cytokines and growth factors on an angiogenesis cytokine array membrane. (**b**) Quantification of relative protein production levels in HUVECs and HAECs. $n = 3$. $^{*}p < 0.05$, $^{**}p < 0.01$ and $^{***}p < 0.001$ *versus* HUVECs or the HUVECs_CM group.

Supplementary Figure 8

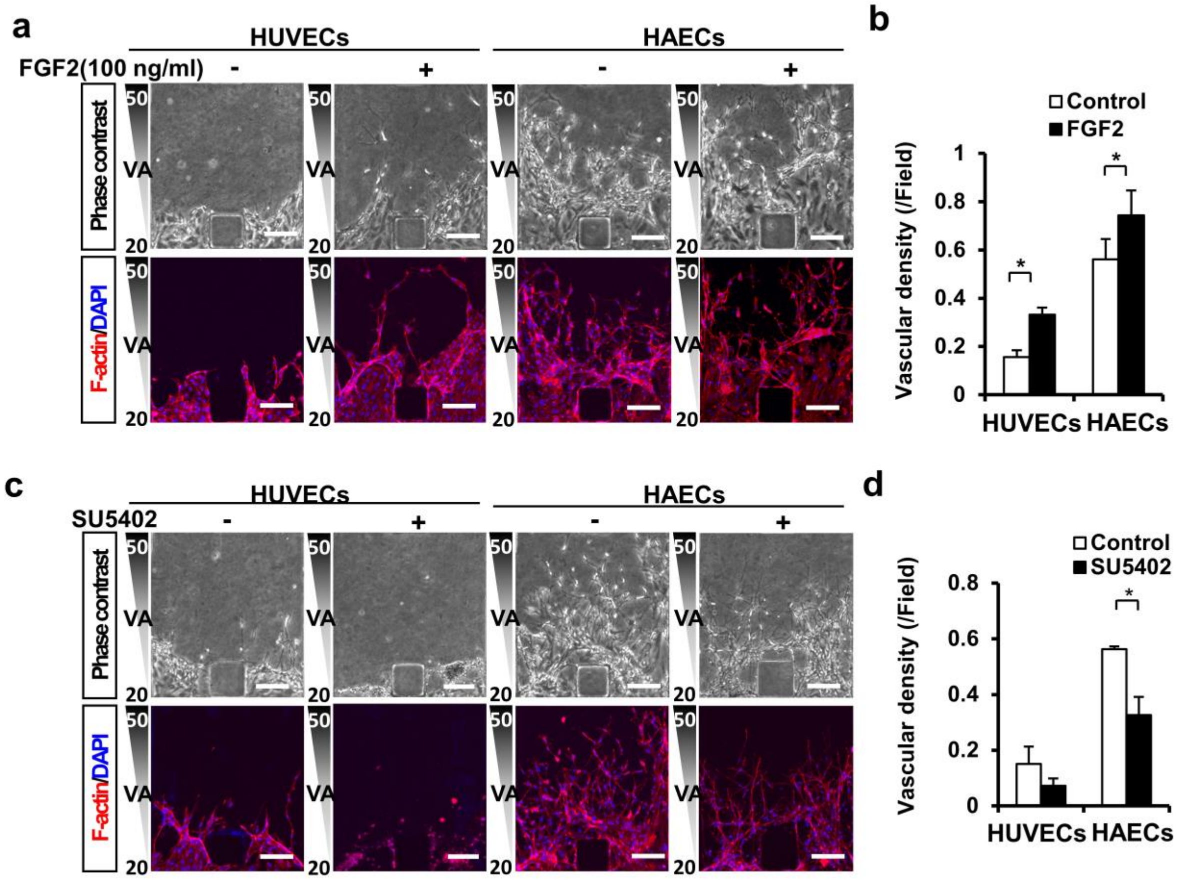

Supplementary Figure 8. FGF2 is a crucial factor for angiogenesis via VEGF-A stimulation. (**a**) Phase contrast image of HUVECs and HAECs in the 3D microfluidic angiogenesis system with the addition of FGF2 (100 ng/ml). Immunofluorescence staining using antibody followed by F-actin (red) and DAPI (blue) staining. Scale bar = 150 μm. (**b**) Quantification the rate of vascular density per field between HUVECs and HAECs. $n = 6$. (**c**) Phase contrast image of HUVECs and HAECs in the 3D microfluidic angiogenesis system with addition of the FGFR inhibitor SU5402 (10 nM). Immunofluorescence staining using antibody followed by F-actin (red) and DAPI (blue) staining. Scale bar = 150 μm. (**d**) Quantification the rate of vascular density per field between HUVECs and HAECs with the addition of SU5402. $n = 6$. $^{*}p < 0.05$ *versus* Control group.

Supplementary Figure 9

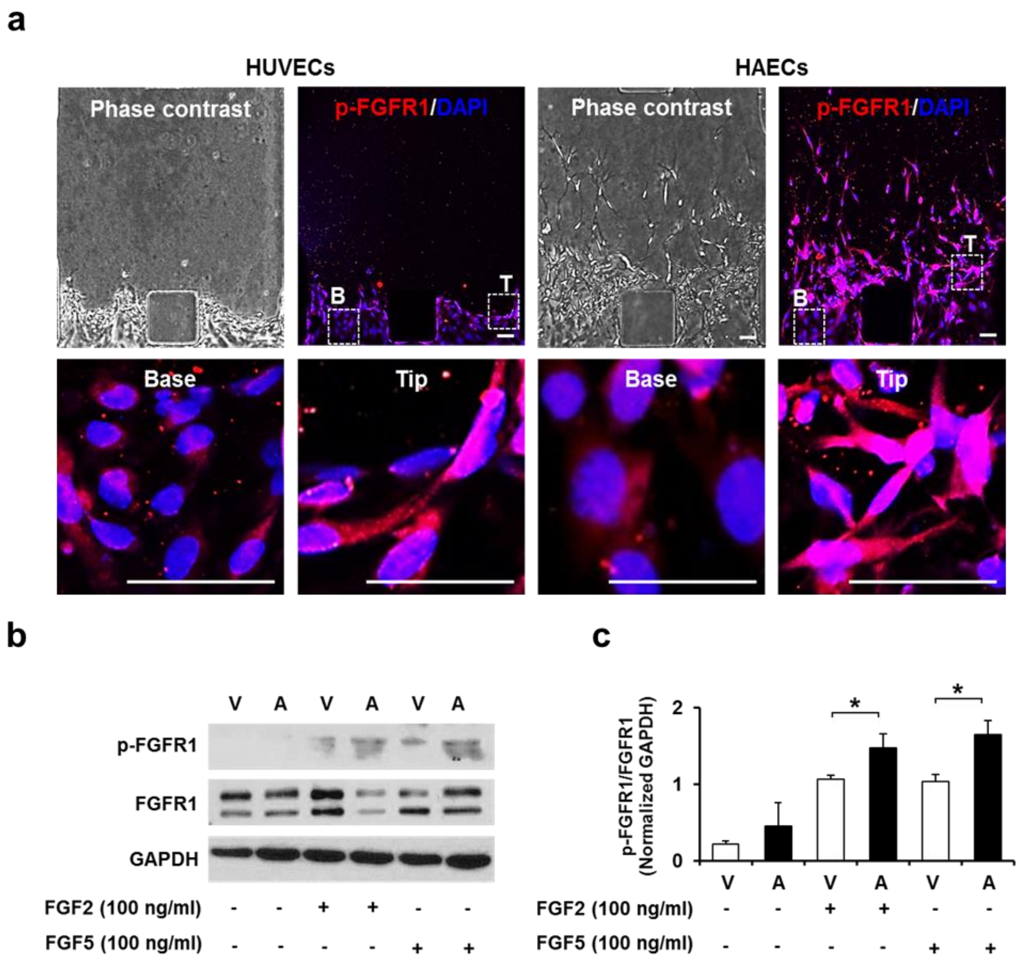

Supplementary Figure 9. HAECs have more FGFR1 sensitivity than HUVECs. (**a**) Phase contrast and immunofluorescence image of HUVECs and HAECs in the 3D microfluidic angiogenesis system. Immunofluorescence staining using antibody followed by p-FGFR1 (red) and DAPI (blue). Scale bar = 50 μm. 'B' indicates base part and 'T' indicates tip part of sprouted vessel. (**b**) HUVECs and HAECs were stimulated for 30 min with basal medium (lane 1 and 2), basal medium containing 100 ng/ml FGF2 (lane 3 and 4) or FGF5 (lane 5 and 6). Total FGFR1 and GADPH were evaluated as a loading control. 'V' indicates HUVECs and 'A' indicates HAECs. (**c**) Activation of FGFR1 was evaluated by quantification of western blot intensity and blotting for p-FGFR1. $n = 3$. $^{*}p < 0.05$ versus HUVECs group.

Supplementary Table 1. List of primers and product sizes

Gene name	Forward sequence (5'-3')	Reverse sequence (5'-3')	Product (bp)
**GAPDH**	ACCACCATGGAGAAGGC	GGCATGGACTGTGGTCATGA	119
**FGF1**	TGAGAAGAAGACACCAAGTGGA	TTGTGGCGCTTTCAAGACTA	110
**FGF2**	AGCGGCTGTACTGCAAAAAC	GCTTGAAGTTGTAGCTTGATGTG	109
**FGF3**	CTACTGCGCCACGAAGTACC	TCCACTGCCGTTATCTCCA	102
**FGF4**	CGGCTCTACTGCAACGTG	GCCGAAGATGCTCACCAC	138
**FGF5**	ACTGGCCAATTTTTGAAATAAGAT	CTGAGACTTTCAAATAGGGCAGA	96
**FGF6**	GGACCCACGAGGAGAACC	TCACTCCAAAGAGACTCACCAC	78
**FGFR1**	CCACCTACTTCTCCGTCAATG	GGGTTTGGTTTGGTGTTATCTG	112
**FGFR2**	TCTGCGTTTGGAGTTGCTC	GCTGCTGCTGCAGTCACTT	124
**FGFR3**	TGGGTTTTCTCATCACTCTGC	CCACCAGGATGAACAGGAAG	134
**FGFR4**	ATTCCATCGGCCTCTCCTAC	TAGCAAAGTGGGAGACTTGGT	121
**VEGFR2**	ATGACATTTTGATCATGGAGC	CCCAGATGCCGTGCATGAG	193
**CXCR4**	CCTGCCTGGTATTGTCATCC	AGGATGACTGTGGTCTTGAGG	105
**GBX2**	AAAGAGGGCTCGCTGCTC	ATCGCTCTCCAGCGAGAA	144
**COL8A1**	CATCTCAAGAACAAAAGACAACTGA	TTGCTGGTGCCTTCCTGT	107
**CXCL10**	GAAAGCAGTTAGCAAGGAAAGGT	GACATATACTCCATGTAGGGAAGTGA	132
**HMOX1**	TGAACTCCCTGGAGATGACTC	AGCTCCTGCAACTCCTCAAA	103
**ID1**	GAATCATGAAAGTCGCCAGTG	ACAGACAGCGCACCACCT	111
**TSPAN12**	CAATGGCCAGAGAAGATTCC	CTGCCAACACACTGATGAC	93
**PLAU**	TTGCTCACCACAACGACATT	GGCAGGCAGATGGTCTGTAT	94
**CCL2**	AGTCTCTGCCGCCCTTCT	GTGACTGGGGCATTGATTG	93
**PTGS2**	CTTCACGCATCAGTTTTTCAAG	TCACCGTAAATATGATTTAAGTCCAC	96
**APOLD1**	CCAGGGGTACTCGGAAGG	AGCAGCAGTCCCTGGAAG	136
**ANGPT2**	TGCCACGGTGAATAATTCAG	TTCTTCTTTAGCAACAGTGGG	123
**HOXA5**	GCGCAAGCTGCACATAAG	CGGTTGAAGTGGAACTCCTT	114
